# Physical Evidence of Oil Uptake and Toxicity Assessment of Amphiphilic Grafted Nanoparticles Used as Oil Dispersants

**DOI:** 10.1021/acs.est.1c08564

**Published:** 2022-05-17

**Authors:** Christopher B. Keller, Hajime Kurita-Oyamada, Scott M. Grayson, Nancy D. Denslow

**Affiliations:** † Department of Chemistry, Tulane University, New Orleans, Louisiana 70118, United States; ‡ Department of Physiological Sciences and Center for Environmental and Human Toxicology, University of Florida, Gainesville, Florida 32611, United States

**Keywords:** toxicology, polymer-grafted nanoparticles, fathead minnow, EROD assay, oil encapsulation

## Abstract

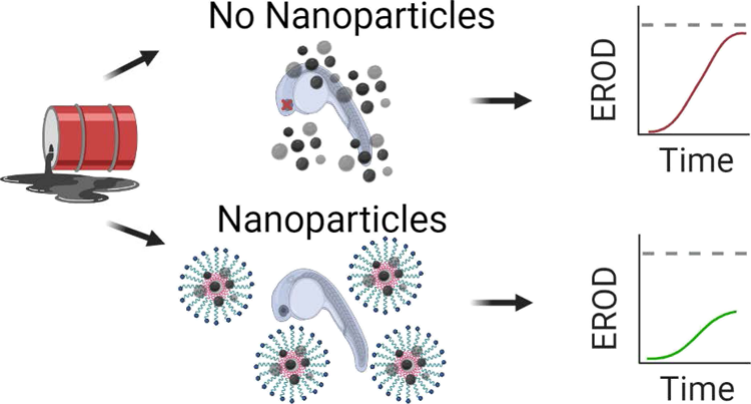

Herein, we report the toxicity evaluation of a new prototype dispersant system, silicon dioxide nanoparticles (NPs) functionalized with (3-glycidoxypropyl)triethoxysilane (GPS) and grafted poly(ε-caprolactone)-*block*-poly[oligo(ethylene glycol)methyl methacrylate mono-methyl ether] (NP-PCL-POEGMA). This serves as a follow up of our previous study where grafted silicon dioxide NPs functionalized with GPS and grafted hyperbranched poly(glycidol) (NP-HPG) were evaluated for reducing the toxicity in embryo, juvenile, and adult fish populations. In this study, the NP-HPG sample is used as a baseline to compare against the new NP-PCL-POEGMA samples. The relative size was established for three NP-PCL-POEGMA samples via cryogenic transmission electron microscopy. A quantitative mortality study determined that these NPs are non-toxic to embryo populations. An ethoxyresorufin-O-deethylase assay was performed on these NP-PCL-POEGMA samples to test for reduced cytochrome P450 1A after the embryos were exposed to the water-accommodated fraction of crude oil. Overall, these NP-PCL-POEGMA NPs better protected the embryo populations than the previous NP-HPG sample (using a protein activity end point), showing a trend in the right direction for prototype dispersants to replace the commercially utilized Corexit.

## Abstract 2

Polymer-grafted nanoparticles are efficient at encapsulating oil and reducing toxicity in fish populations.

## Introduction

In the last decade, the Gulf of Mexico was the focus of attention after the Deepwater Horizon pipe explosion with the catastrophic environmental damage caused by the event still under investigation.^1^ The current strategy for the treatment of oil spills is the use of chemical dispersants (e.g., Corexit).^2,3^ However, several studies have demonstrated that this type of remediation makes the components of oil more bioavailable to fish and other aquatic organisms.^4−8^ As a result, a strong effort has been made to generate alternative methods to clean up the oil spills, ultimately replacing Corexit.^9−17^

In our previous publication,^18^ we discussed a novel alternative to remediate oil spills. Polymer-modified silica nanoparticles (NPs) were synthesized where the inner regime of the grafted polymer is hydrophobic and the outermost portion is hydrophilic, thereby creating room for the entrapment of oil while keeping the particles suspended under aqueous conditions. This configuration was formed by grafting amphiphilic polymer branches, with known biocompatibility and stability. We demonstrated this concept by grafting hyperbranched poly(glycidol) as a chemical branch^13^ from the surface of (3-glycidoxypropyl)triethoxysilane (GPS)-functionalized silica NPs. GPS functionalization was determined to be a necessary step as no further grafting reactions were successful without increased hydroxyl sites on the silica NP surface. Biocompatibility and efficacy were probed by exposing the NP-hyperbranched poly(glycidol) (NP-HPG) sample to fathead minnow (FHM) embryonic and juvenile stages, either NP alone or in combination with oil components. This experiment showed promising results toward the usage of this nanomaterial as an oil dispersant.^18^ In this study, we present a follow-up study where the same concept is applied but with poly(caprolactone) poly[oligo(ethylene glycol)methyl methacrylate mono-methyl ether] (PCL-*b*-POEGMA) polymeric branches. PCL, a linear internal hydrophobic polymer, and POEGMA, the branched outermost hydrophilic polymer, are grafted from GPS-functionalized silica NPs. Three NP-PCL-POEGMA samples are evaluated herein for further evidence of efficacy of these NPs to remediate an oil spill by examining the direct physical evidence of oil entrapment. Toxicity analysis was additionally carried out by measuring the enzymatic activity of the previously studied cytochrome P450 1A (CYP1A) gene. To do this, the ethoxyresorufin-O-deethylase (EROD) assay was performed on whole FHM embryos to measure the efficacy of oil component entrapment.

## Materials and Methods

### NP-PCL-POEGMA NPs

Three NP-PCL-POEGMA samples were synthesized as previously reported.^19,20^ Complete characterization of these samples can be found in Keller et al.^20^ Henceforth, these samples will be referred to as NP-*X*-*Y*, corresponding to the labeling system used in Keller et al., where *X* and *Y* represent the degree of polymerization for PCL and POEGMA, respectively. The degree of polymerization is calculated from the number-average molecular weight (*M*_n_) taken from GPC measurements. A brief overview of the chemical composition for all the three NP-PCL-POEGMA samples is outlined in Table 1. The silica (SiO_2_) core was provided by Nissan Chemical Ltd. with an average size of 100–150 nm in diameter (from TEM analysis).

**Table 1:** Composition of NP-PCL-POEGMAs^20^

	% PCL (w/w)	% POEGMA (w/w)	% SiO_2_ core(w/w)	*M*_n_ PCL_GPC_ (Da)	*M*_n_ POEGMA_calc_ (Da)	OEGMA/ε-caprolactone^a^
NP-54-15	27.3	31.5	41.2	6200	7400	0.27
NP-46-36	17.2	54.3	28.6	5300	18 100	0.78
NP-54-47	16.9	57.5	25.6	6200	23 300	0.86

a The ratio of OEGMA monomer to ε-caprolactone monomer representing the approximate ratio of hydrophilic to hydrophobic content calculated from experimental TGA measurements.

### Transmission Electron Microscopy

Images were collected on a FEI TECNAI G2F30 instrument. Cryogenic transmission electron microscopy (cryo-TEM) experiments were performed for the following study at 200 kV. Three NP-PCL-POEGMA samples, prior to oil exposure, were prepared at a concentration of 1 mg mL^–1^ in deionized water. Anadarko crude oil (100 μL) was added directly to 500 μL of 1 mg mL^–1^ aqueous solution of suspended NPs and stirred at 1400 rpm for 24 h. After 24 h of exposure time, 30 μL of the aqueous phase (containing the suspended NPs) was removed via a glass, gas-tight syringe and diluted with 30 μL of deionized water. Utilizing a FEI Vitrobot, 5 μL of the sample was measured via a micropipette and placed on a lacey carbon 200 mesh copper TEM grid. The grid was then plunged into liquid ethane after being sufficiently blotted once by filter paper for 1.5 s at 100% humidity. After vitrifying the three samples, they were transferred to a cryogenic TEM holder and held at −170 °C.

### Toxicity Using FHM Experiments

FHM (*Pimephales promelas*) embryos were acquired from Aquatic Biosystems Inc. The package was delivered with an ice pack to delay the development of embryos. At the time of exposure, the embryos were at the pigmented eye stage and chemically dechorionated using 50 mg mL^–1^ pronase.^21^ Briefly, approximately 300 embryos with pigmented eyes were placed in a crystallization dish with a minimum of water. Then, 500 μL of 50 mg mL^–1^ pronase was added to the dish containing the embryos. The dish was gently agitated for better pronase dispersion and left to react for 3 min. After the digestion time, the embryos were transferred immediately to a 1 L beaker containing clear water and rinsed thoroughly three times or until all chorions were removed. Dechorionated fish were transferred to a clean container for sorting into beakers used in exposure treatments. All animal experiments were conducted in strict accordance with the protocols approved by the University of Florida IACUC.

### Exposure Design

All embryo exposures were performed in quadruplicate and were carried out using a small-volume beaker (∼20 mL). Ten embryos were exposed in each beaker. The exposure solutions were changed by 50% every day, and the duration of the exposure was 96 h. In all cases, glassware was used in the entire process. All testing solutions were prepared using reconstituted fresh water with 20% Hanks solution (27 mM NaCl, 1 mM KCl, 0.05 mM Na_2_HPO_4_, 0.09 mM KH_2_PO_4_, 0.25 mM CaCl_2_, 0.2 mM MgSO_4_, and 41 mM NaHCO_3_ in Milli-Q water). Hanks solution (20%) acted as the negative control. To test the efficacy and toxicity of the NPs, the water-accommodated fraction (WAF) of crude oil was used. The Macondo crude oil used was provided by AECOM Environment Toxicology Laboratory (collected from the leaking well from Deepwater Horizon British Petroleum). The WAF was prepared by mixing 1 mL of oil in 1 L of 20% Hanks solution and weathered for 1 week by mixing the solution in a hood with a stir bar.^4^ At the 1 week timepoint, the mixture was settled using a separatory funnel, and then the aqueous phase was separated from the oil. The Macondo oil and the WAF were characterized for their constituents by Columbia Analytical Services (Kelso, WA) through analyses of PAH, BTEX, and low-molecular-weight PAHs. HCl (1 mL) was added to 1 L of WAF as a preservative prior to shipping the samples to the laboratory. The chemical composition of each is reported in Table S2.

For the solutions containing NPs, the concentration of NPs dispersed in water was 20 mg L^–1^. Testing solutions were prepared by spiking the NP-PCL-POEGMA and NP-HPG NP stock solutions into 20% Hanks solution. This was performed for all the four NP samples independently. To keep the particles suspended, each concentration of exposure solution (including the Hanks control) was aerated with a constant flow of air, forcing the movement of liquid inside the exposure beakers.

### Toxicity Evaluation via the EROD Assay

The mortality and temperature were checked daily, and any embryos that died were removed. This experiment involved exposing the FHM embryos to a combination of NPs and WAF as described in the above section.

For the EROD assay, after 96 h of WAF and WAF + NP exposure, the remaining embryos were transferred into wells containing 7-ethoxy resorufin (7-ER) (CAS 5725-91-7) at 0.36 μg mL^–1^ to test for the enzymatic activity of CYP1A enzyme. A positive control was prepared by dissolving β-naphthoflavone (B-NF, 75 μg L^–1^) (Sigma-Aldrich, CAS 6051-87-2, purity ≥ 98%) in 20% Hanks solution using a modified protocol by Boehler et al.^22^ Each embryo was transferred to a pre-filled well in a 96-well plate.

To optimize the incubation time for the assay in 7-ER, different time lengths were evaluated using four different plates at 30 min increments of time, starting with 30 min up to 120 min. The EROD activity was measured using each individual fish’s media (200 μL) by transferring it to a new clear-bottom plate for the measurement of resorufin, the byproduct of the metabolization of 7-ER by CYP1A. After the incubation time, the assay was completed with fluorescence readings. The fluorescence acquisition parameters were excitation at 535 nm and detection at 590 nm.

### General Toxicity

To quantify the toxic effects of WAF and WAF + NP on fish, the mortality was checked every day over 96 h. Death of embryos was determined if no blood flow could be observed under the microscope or when fish looked opaque through observation using a transilluminator.

### Statistical Analysis

EROD results were analyzed and plotted using R software. To statistically determine the significance of the observed differences, a linear model was used. All treatments, time intervals, and plates were considered in the model. To compare only those treatments that contained the WAF, treatments with Hanks and B-NF were excluded from the analysis.

## Results and Discussion

### NP Size Evaluation

NP-PCL-POEGMAs were reported previously in the literature with the ability to encapsulate crude oil.^19,20^ In order to fully understand the real-world effects from the utilization of these NPs in water environments, their toxicity needs to be investigated. Given that these NPs are dispersed in large bodies of water during their application, exposure to aquatic populations is inevitable. As such, a toxicity evaluation is critical to understanding the broader impact in these systems.

**Figure 1:**
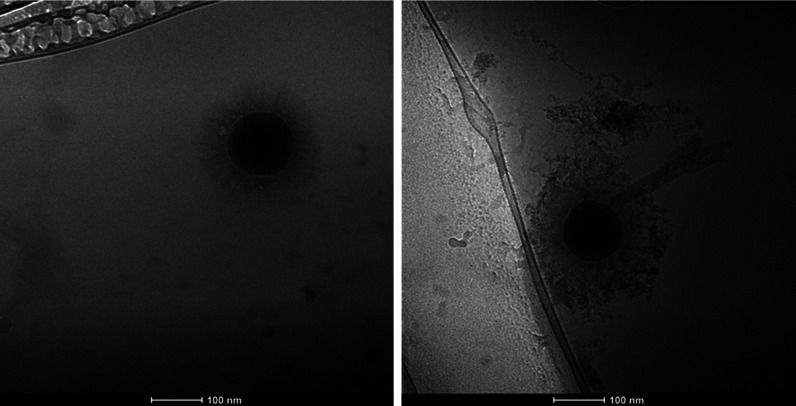
Cryo-TEM of NP-54-15 before (left) and after (right) exposure to Anadarko crude oil. The scale bar is 100 nm.

One of the most asked questions for these NP systems is the overall size of the final particles, which is thought to be an important parameter for toxicity evaluation. As a method to establish the size of these NPs, cryogenic TEM imaging was performed, while the NPs were dispersed under aqueous conditions (Figures 1, S1, and S2). Three NP-PCL-POEGMAs, previously reported in the literature, were analyzed under cryogenic TEM conditions to study the size changes in aqueous environments, both before and after exposure to Anadarko crude oil (Table 2). The values reported are averaged between three to four particles per sample studied, with standard deviations. For the purpose of initial investigation, the samples were dispersed in deionized water. This was done to generalize the behavior of these NPs after encapsulation of hydrophobic molecules.

**Table 2:** Cryo-TEM Size Measurements of All the Three NP-PCL-POEGMAs before and after Exposure to Anadarko Crude Oil

sample	average total diameter prior to oil exposure (nm)	average total core diameter prior to oil exposure (nm)	average total diameter after oil exposure (nm)	average total core diameter after oil exposure (nm)	Δ (nm)
NP-54-15	246.7 ± 3.8	119.0 ± 2.7	279.0 ± 15.1	121.0 ± 2.5	32.3
NP-46-36	338.9 ± 11.7	125.4 ± 5.7	344.8 ± 2.7	135.8 ± 2.2	5.9
NP-54-47	298.4 ± 4.4	123.8 ± 0	305.6 ± 10.9	119.0 ± 1.0	7.2

On average, NP-54-15 had a diameter of 246.7 ± 3.8 nm prior to oil exposure. These NP-PCL-POEGMAs were exposed to crude oil over a period of 24 h and afterward allowed to equilibrate for 2 days. The average diameter of NP-54-15 increased from 246.7, before oil exposure, to 279.0 ± 15.1 nm, after exposure to Anadarko crude oil, representing an increase of 32.3 nm. However, the polymer brush length (Table 3) for NP-54-15, where the contribution from the NP core is removed, increased from 63.8 nm (before oil exposure) to 79.0 ± 6.3 nm (after oil exposure), a difference of approximately 15.2 nm. These NPs have been designed in such a way that without the presence of any hydrophobic molecule (e.g., oil), the PCL chains are partially collapsed. Once hydrophobic molecules are introduced and diffused into the PCL block, the chains expand, increasing the total polymer brush length. The observation of this phenomenon confirms the presence of oil within the grafted polymer chains of NP-54-15.

**Table 3:** Polymer Brush Lengths Calculated from the Total Average Radius Measured from Cryo-TEM Images Once the Radius of the SiO_2_ Core Was Subtracted

sample	brush length prior to oil exposure (nm)	brush length after exposure to oil (nm)	Δ (nm)
NP-54-15	63.8 ± 0.6	79.0 ± 6.3	15.2
NP-46-36	106.7 ± 5.2	104.5 ± 0.9	–2.2
NP-54-47	87.3 ± 2.2	92.8 ± 5.0	5.5

This evaluation was repeated for the remaining two samples. NP-46-36 was found to have a diameter of 338.9 ± 11.7 nm prior to oil exposure (Figure S1). After exposure to Anadarko crude oil, the diameter of NP-46-36 increased to 344.8 ± 2.7 nm. Although this appears to fit the trend established by NP-54-15 (stemming from the increase in diameter of 5.9 nm), a subtraction of the silica NP core (the average of three to four particles per sample was measured as 125.4 nm before exposure and 135.8 nm after exposure) reveals that the calculated polymer brush length (Table 3) decreased by approximately 2.2 nm. Further, the ratio of polymer brush length to silica NP core was calculated to decrease from 1.7:1 prior to oil exposure to 1.5:1 after oil exposure (Table S1). NP-46-36 depicts the only observed sample to have a decrease in average particle size. This is perhaps an argument of polymer dispersity and silica core size. However, it is important to note that oil encapsulation was confirmed via other analyses.

Encapsulation studies conducted with UV absorbance previously confirmed the presence of oil encapsulation for all the three NP-PCL-POEGMA samples.^20^ The cryo-TEM values reported in this study are averaged values taken from a series of NPs from each sample. As mentioned, it may be that the NPs imaged after oil encapsulation had lower overall polymer chain length than the NPs selected prior to oil encapsulation. The size of the silica NP core was on average 10.4 nm larger after oil encapsulation than before oil exposure, given that we know that the silica starting materials are reported as a range (100–150 nm), this perhaps is the source of the deviation from the expected result. These samples were additionally processed via sonication to break up aggregates before cryo-TEM studies, which may contribute to randomized cleavage of polymer chains given the high energy input. With several variables potentially contributing to the observed differences in particle size, it is difficult to definitively state from where this deviation from an increase in size after oil encapsulation originates.

NP-54-47 interestingly mimicked the behavior seen in NP-54-15, both coming from the same parent SiO_2_-GPS-PCL NP precursor. Prior to oil exposure, NP-54-47 had a diameter of 298.4 ± 4.4 nm (Figure S2). After exposure to Anadarko crude oil, the diameter was 305.6 ± 10.9 nm (Figure S2), an increase of 7.2 nm. The total polymer brush length for NP-54-47 increased by approximately 5.5 nm after the uptake of oil, confirming the PCL chain expansion upon oil uptake. It is important to note that when comparing the relative ratios of the PCL block to the POEGMA block within these three NP-PCL-POEGMA samples, NP-46-36 and NP-54-47 contained roughly the same ratio of PCL to POEGMA per repeat unit (0.78 and 0.86 OEGMA:ε-caprolactone, respectively). In contrast, NP-54-15 had more PCL than POEGMA per repeat unit at an OEGMA:ε-caprolactone ratio of 0.27. Evaluation of the polymer chain expansion after oil exposure revealed that increased POEGMA block perhaps hinders the diffusion of oil into the PCL block, resulting in lower increases in NP diameter overall.

### NP Toxicity

Following the size evaluation of the three NP-PCL-POEGMA NPs, the toxicity and efficacy of the NPs exposed to the embryonic stage of FHM were critically investigated by looking for mortality and through the EROD assay, respectively. Mortality evaluation was performed to generalize the effects of exposure to the three NP-PCL-POEGMA samples on the embryo fish population. In order to establish a control, NP-HPG from the previous study^18^ was recorded alongside the study of the NP-PCL-POEGMAs. No mortality was observed in any treatment, suggesting that these combinations of NPs and oil are safe at the studied concentration (20 mg L^–1^).

An adaptation of the EROD assay^23^ was performed to measure the enzymatic activity of CYP1A. For this, FHM embryos were exposed to the WAF of crude oil, containing polyaromatic hydrocarbons (PAHs) that promote the expression of the CYP1A protein and its activity. The exposure occurred over a 24 h period. In addition to WAF alone, the FHM embryos were treated with WAF plus NPs to investigate if the addition of NPs ameliorated the effects of the WAF.

**Figure 2:**
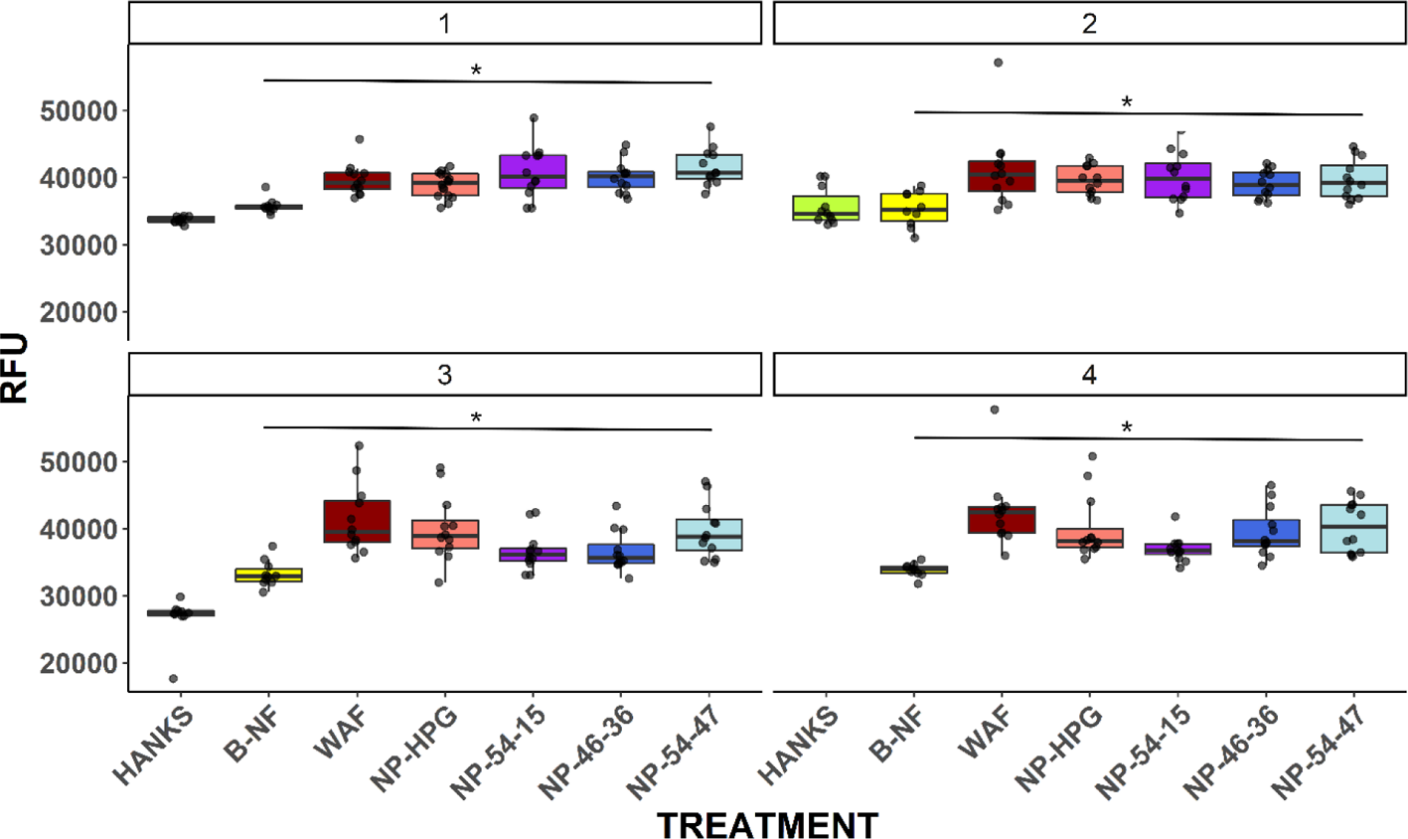
Observed fluorescence after the EROD assay using plates with different incubation times: plate 1: 30 min, plate 2: 60 min, plate 3: 90 min, and plate 4:120 min. Boxes represent the 75th and 25th quartile range, and each dot is an individual fish value. The horizontal line in the box represents the median. Treatments: negative control, Hanks; positive control for EROD, B-NF; positive control for oil, WAF; NP treatments with WAF (WAF + NPs), NP-HPG, NP-54-15, NP-46-36, and NP-54-47. The asterisk denotes the statistical difference (*p* < 0.05) when compared to Hanks, except in plate 4, where the comparison was against B-NF.

Figure 2 depicts the observed relative fluorescence readings for the four plates after different incubation times. All treatments with WAF significantly induced EROD independently of the incubation time, with higher relative fluorescence compared to the Hanks control. While resorufin could be quantified within the first hour of incubation (plates 1 and 2), the assay was not fully optimized until 90–120 min of incubation. Plates 3 and 4 revealed greater differences between NP-PCL-POEGMA samples for all treatments compared to WAF alone, indicating that the differences in CYP1A activity can be better assessed after 90 min of incubation (Figure 2, plates 3 and 4).

**Figure 3:**
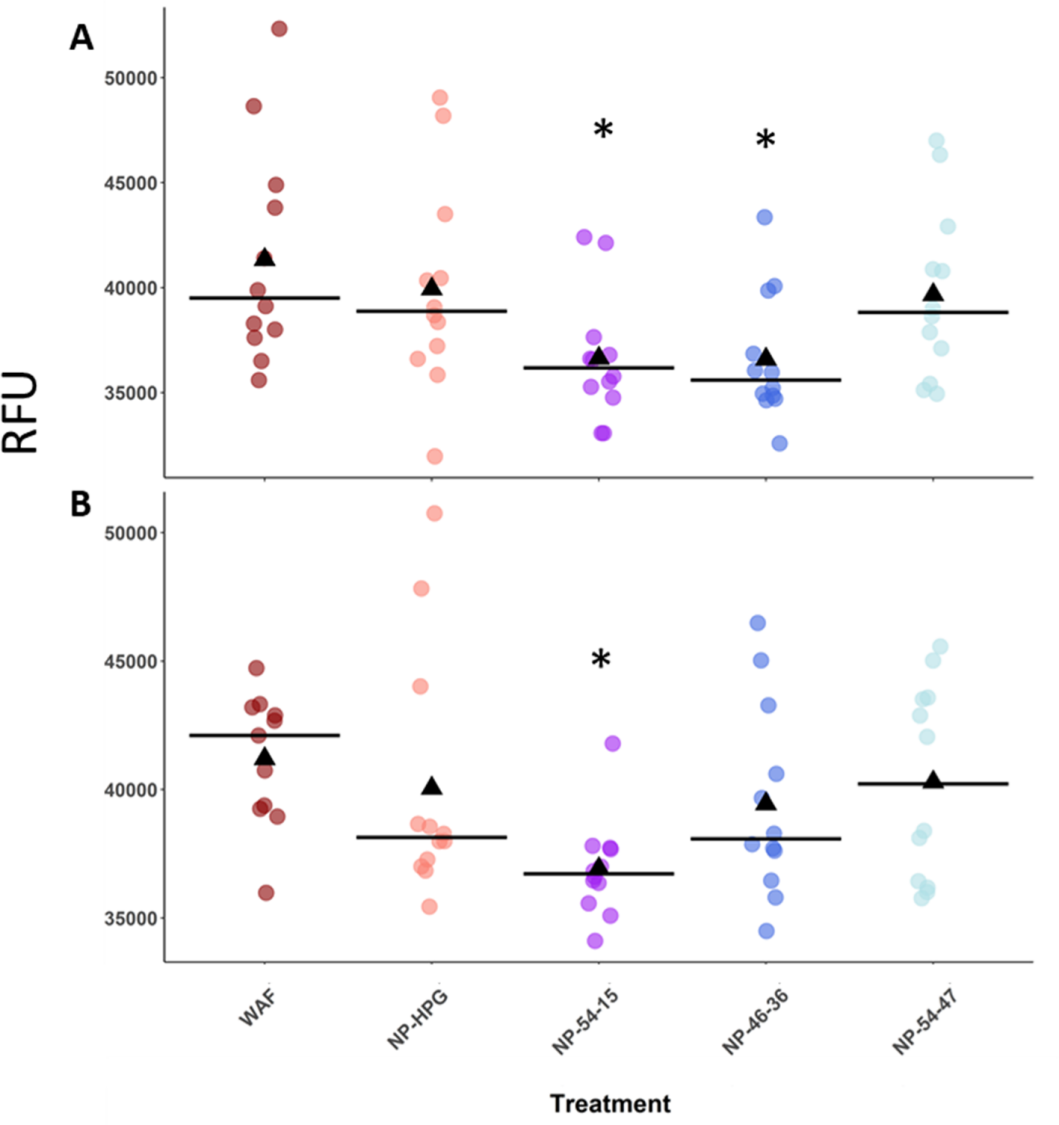
Analysis of the development of the resorufin reading comparing the effect of the addition of NPs to WAF after (A) 60 and (B) 90 min. The triangles represent the median values and the crossbars represent the mean. (A) Plate 3 and (B) plate 4. The asterisks denote the significant difference when compared to WAF.

Figure 3 illustrates the individual fish values obtained for the optimized assays (plate 3 and plate 4) where the relative fluorescence of WAF alone is compared to the treatments with WAF + NP.

After analyzing with the non-parametric test of Kruskal–Wallis, the WAF + NP treatments in plate 3 and 4 were significantly different from WAF alone, thereby reinforcing the hypothesis that the NPs are effectively reducing the expression of CYP1A in fish embryos. In plate 3, it can be observed that both NP-54-15 and NP-46-36 had the lower median values (black triangles) among the treatments after Dunnett’s post hoc test. Similarly, in plate 4, the treatment containing NP-54-15 was significantly different after Dunnett’s test. A generalized linear model applied to all plates, combining the different incubation times, also demonstrated that the mean values of NP-54-15 had significantly lowered relative fluorescence units. Thus, NP-54-15, which had the largest increase of polymer brush length after oil exposure, showed the greatest decrease in mean fluorescence values; reaffirming the higher levels of oil uptake by these particles results in lower overall fluorescence in the EROD test. In our previous report, we found that NP-HPG NPs lowered the expression of CYP1A mRNAs in juvenile stages of FHM. There is likely a temporal difference between induction of CYP1A mRNA and activity of CYP1A protein, as measured by EROD.

The EROD assay is broadly used as a biomarker of exposure to planar halogenated/polycyclic aromatic hydrocarbons.^24^ Low to intermediate molecular weight PAHs (e.g., less than 8–10 rings) are known to be soluble in water, with intermediate molecular weight PAHs persisting even after low molecular weights have volatilized.^25−33^ Therefore, the number of hydrocarbons readily available for encapsulation is directly correlated with the hydrocarbons solubilized within the aqueous phase. As detailed in Table S1, both the crude oil and the WAF solution contained low molecular weight PAHs (naphthalene, phenanthrene, anthracene, pyrene, benzo[*a*]pyrene, etc.). The EROD assay thus acts as an indirect indication of how decreased PAH, stemming from NP encapsulation, can result in lower CYP1A activity. This assay is generally assessed using the microsomal portion of a homogenate of fish liver.^34^ Various other authors have shown that this enzymatic activity can be measured in the whole body of juvenile and larval stages of fish.^22,23,35−37^ Several species of fish have been assessed for whole-body EROD assay, such as zebrafish,^38^ rainbow trout,^39^ mummichogs,^23^ medaka,^40^ and FHM.^22^ Here, we use FHM embryos that have been dechorionated. Digestion of the chorion facilitates the diffusion of the byproduct of the enzymatic activity of CYP1A to the culture media. The embryonic stage was chosen for this study because they fit best within the dimensions of a 96-well plate, which are not big enough to accommodate a juvenile FHM. Utilization of a 96-well plate afforded a high-throughput method for evaluating the biomarker of exposure. Kais et al. using zebrafish larvae demonstrated that in the early stages of development, the CYP1A activity can be observed under fluorescence microscopy, first in the brain, eyes, and otoliths.^37^ Thus, we anticipated the same pattern in FHM and that the end product (resorufin) would diffuse into the media. In this study, we used embryos that were ∼2 days post-fertilization (dpf) with pigmented eyes and successfully demonstrated a measurable fluorescence of resorufin. The earliest whole FHM developmental stage with EROD activity we found in the literature was after hatching.^22^

With this novel approach, we have demonstrated that prehatch FHM can already metabolize oil components, and the product resorufin can be measured as early as 2 dpf. Additionally, we demonstrated that an incubation period of greater than 60 min allows the diffusion of resorufin, for better resolution. Further, the addition of NP-PCL-POEGMA to WAF can ameliorate the effects of oil exposure on FHM by decreasing the bioavailability of PAHs in WAF. This was considered to be highly significant since there are many publications suggesting that the current chemical dispersant, Corexit, does the opposite and increases the bioavailability of PAHs in oil to surrounding organisms.^4,41−43^ As hypothesized, these new NP-PCL-POEGMAs perform better than the previously reported NP-HPG particles. These improved particles appear to function well, concerning the dispersion of oil and reducing the bioavailability of PAHs to aquatic organisms. The results from these experiments give us a better perspective on how well these engineered NPs may function, as we continue the research to improve the ability of NPs to encapsulate oil.

## Supporting Information Available

Cryo-TEM data for NP-46-36 and NP-54-47, ratio of polymer brush length before and after exposure to Anadarko crude, and chemical analysis of Macondo crude oil and WAF of the oil (PDF)

## Abbreviations

NPs: nanoparticles
HPG: hyperbranched poly(glycidol)
PCL-POEGMA: poly(caprolactone)-*block*-poly[oligo(ethylene glycol)methyl methacrylate mono-methyl ether]
Cryo-TEM: cryogenic transmission electron microscopy
EROD: ethoxyresorufin-O-deethylase
WAF: water-accommodated fraction
CYP1A: cytochrome P450 1A
B-NF: β naphthoflavone
AGNs: amphiphilic grafted nanoparticles
FHM: fathead minnow
RFU: relative fluorescence unit
SiO_2_: silicon dioxide
GPS: (3-glycidoxypropyl)triethoxysilane
PAH: polyaromatic hydrocarbon
