# Supplementary material for: Physical Evidence of Oil Uptake and Toxicity Assessment of Amphiphilic Grafted Nanoparticles Used as Oil Dispersants
Source: Environ Sci Technol. 2022 May 17;56(12):7917–23. doi: 10.1021/acs.est.1c08564 (PMC9227714; doi:10.1021/acs.est.1c08564)
Supplement: Supplementary file 1 — es1c08564_si_001.pdf [file es1c08564_si_001.pdf]

Supplementary Material

Physical evidence of oil uptake and toxicity assessment of amphiphilic grafted nanoparticles used as oil dispersants

Christopher B. Keller[a], Hajime Kurita-Oyamada[b], Scott M. Grayson[a], Nancy D. Denslow[b*]*

[a]Department of Chemistry, Tulane University, New Orleans, LA, 70118, United States

[b]Department of Physiological Sciences and Center for Environmental and Human Toxicology, University of Florida, Gainesville, FL, 32611, United States

*Corresponding authors

Nancy D. Denslow
Department of Physiological Sciences and Center for Environmental and Human Toxicology
University of Florida
PO Box 110885
Gainesville, FL 32611
ndenslow@ufl.edu

Dr. Scott M. Grayson
Department of Chemistry, Percival Stern Hall, Tulane University, New Orleans, Louisiana, USA
E-mail: sgrayson@tulane.edu
Fax: (+1) 504-865-5596

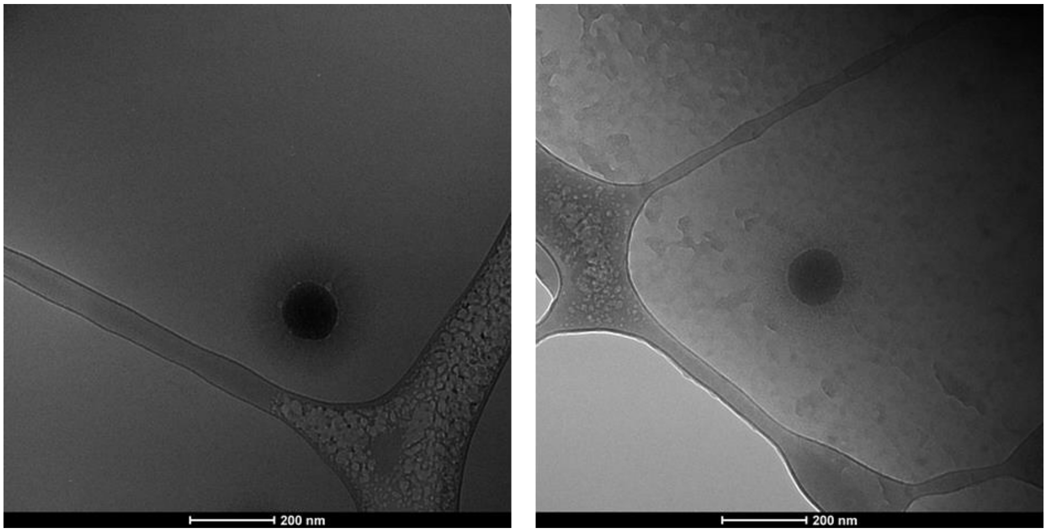

Figure S1. Cryo-TEM of NP-46-36 before (left) and after (right) exposure to Anadarko crude oil. The scale bar is 200 nm.

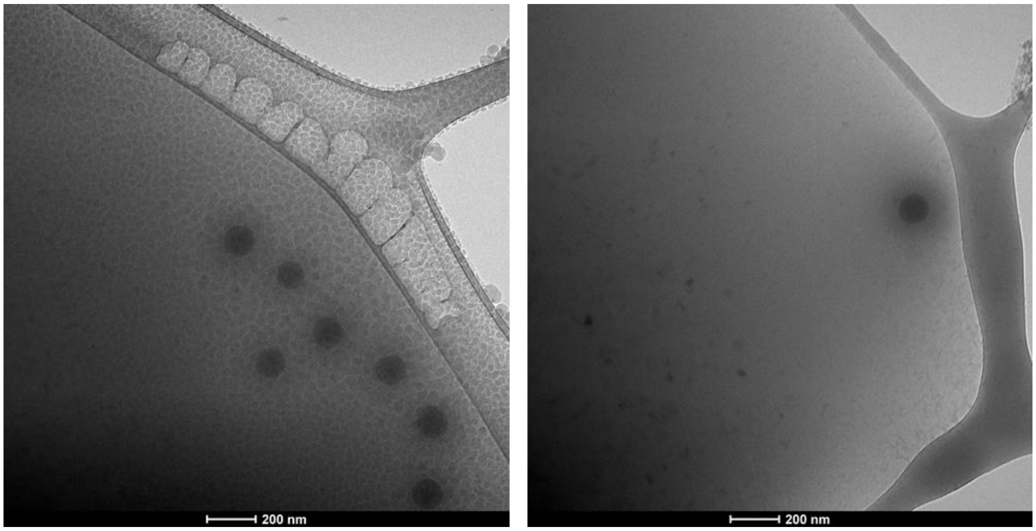

Figure S2. Cryo-TEM of NP-54-47 before (left) and after (right) exposure to Anadarko crude oil. The scale bar is 200 nm.

Table S1. A Ratio of Polymer Brush Length to SiO_2 NP Core before and after exposure to Anadarko crude oil for Samples 1-3.

Sample	*Prior to oil exposure*	*After exposure to oil*
NP-54-15	1.07	1.31
NP-46-36	1.70	1.54
NP-54-47	1.41	1.56

Table S2. Chemical analysis of Macondo crude oil and the water accommodated fraction of the oil (WAF). The table is divided by PAH and alkylated homologues, plus the BTEX species.

PAH & Alkalated homologues (µg/L)	BP Crude Oil (µg/L) neat	WAF-Oil- (µg/L)	PAH & Alkalated homologues (µg/L)	BP Crude Oil (µg/L) neat	WAF-Oil- (µg/L)	BTEX (VOC) (µg/L)	BP Crude Oil (µg/L) neat	WAF-Oil- (µg/L)
cis/trans-Decalin	540	0.331	C4-Fluoranthenes/Pyrenes	86	2.41	Benzene	2700	72
C1-Decalins	880	0.604	Naphthobenzothiophene	15	0.293	Toluene	7300	39
C2-Decalins	1100	1.58	C1-Naphthobenzothiophenes	57	1.34	Ethylbenzene	960	8.4
C3-Decalins	1100	3.98	C2-Naphthobenzothiophenes	69	1.45	m,p-Xylenes	5100	42
C4-Decalins	820	6.58	C3-Naphthobenzothiophenes	58	1.16	o-Xylene	1800	21
Benzo(b)thiophene	8.1	0.0849	C4-Naphthobenzothiophenes	27	0.641	Styrene		ND
C1-Benzothiophenes	29	0.629	Benz(a)anthracene	6.2	0.144	Isopropylbenzene		0.81
C2-Benzothiophenes	23	1.15	Chrysene	44	0.818	n-Propylbenzene		1
C3-Benzothiophenes	27	0.769	C1-Chrysenes	100	2.04	1,3,5-Trimethylbenzene		2.5
C4-Benzothiophenes	28	0.804	C2-Chrysenes	130	2.44	tert-Butylbenzene		ND
Naphthalene	640	127	C3-Chrysenes	94	1.87	1,2,4-Trimethylbenzene		7.8
C1-Naphthalenes	1400	79.6	C4-Chrysenes	74	1.49	sec-Butylbenzene		0.13
C2-Naphthalenes	1800	44.1	Benzo(b)fluoranthene	4.7	0.115	4-Isopropyltoluene		0.15
C3-Naphthalenes	1100	20.3	Benzo(k)fluoranthene	ND	ND	n-Butylbenzene		0.2
C4-Naphthalenes	610	10.2	Benzo(a)fluoranthene	ND	ND	Naphthalene		7.9
Biphenyl	180	7.37	Benzo(e)pyrene	9.6	0.199			
Dibenzofuran	ND	ND	C30-Hopane	61	1.33	Dibromofluoromethane (%)	73	104
Acenaphthylene	ND	ND	Benzo(a)pyrene	1.7	0.0377	Toluene-d8 (%)	85	120
Acenaphthene	12	0.622	Perylene	ND	ND	4-Bromofluorobenzene (%)	56	108
Fluorene	90	5.4	Indeno(1,2,3-cd)pyrene	ND	ND			
C1-Fluorenes	210	6.58	Dibenz(a,h)anthracene	1.9	0.0443			
C2-Fluorenes	280	7.74	Benzo(g,h,i)perylene	1.7	0.0363			
C3-Fluorenes	260	6.73	4-Methyldibenzothiophene	55	1.67			
Anthracene	8.2	0.437	2-Methyldibenzothiophene	25	0.848			
Phenanthrene	200	8.52	1-Methyldibenzothiophene	14	0.571			
C1-Phenanthrenes/Anthracenes	450	14.4	3-Methylphenanthrene	94	3.23			

C2-Phenanthrenes/Anthracenes	470	13.4	2-Methylphenanthrene	110	3.41	
C3-Phenanthrenes/Anthracenes	320	8.5	2-Methylanthracene	4.9	0.287	
C4-Phenanthrenes/Anthracenes	210	6.61	9-Methylphenanthrene	130	4.07	
Retene	9.5	0.243	1-Methylphenanthrene	94	3.02	
Dibenzothiophene	31	1.57	2-Methylnaphthalene	1300	77.2	
C1-Dibenzothiophenes	100	3.91	1-Methylnaphthalene	950	63.6	
C2-Dibenzothiophenes	160	4.96	2,6-Dimethylnaphthalene	870	18.8	
C3-Dibenzothiophenes	120	3.92	2,3,5-Trimethylnaphthalene	350	6.54	
C4-Dibenzothiophenes	63	2.3	Carbazole	ND	1.56	
Benzo(b)fluorene	14	0.362	Fluorene-d10 (%)	86	110	
Fluoranthene	3.7	0.124	Fluoranthene-d10 (%)	107	102	
Pyrene	15	0.388	Terphenyl-d14 (%)	88	116	
C1-Fluoranthenes/Pyrenes	57	1.6	Di(propylene glycol) Butyl Ether	3	12	
C2-Fluoranthenes/Pyrenes	93	2.88	2-Fluorobiphenyl (%)	63	62	
C3-Fluoranthenes/Pyrenes	120	3.35				

The analysis was done by the Columbia Analytical Services (Kelso, WA).
